# The Role and Molecular Mechanism of Non-Coding RNAs in Pathological Cardiac Remodeling

**DOI:** 10.3390/ijms18030608

**Published:** 2017-03-10

**Authors:** Jinning Gao, Wenhua Xu, Jianxun Wang, Kun Wang, Peifeng Li

**Affiliations:** 1 Center for Developmental Cardiology, Institute for Translational Medicine, Qingdao University, Dengzhou Road 38, Qingdao 266021, China; gjn.1127@163.com (J.G.); wangjx@qdu.edu.cn (J.W.); 2 Department of Basic Medical College, Qingdao University Medical College, Ningxia Road 308, Qingdao 266071, China; qd.wh@163.com

**Keywords:** non-coding RNA, microRNA, long non-coding RNA, circular RNA, cardiac remodeling

## Abstract

Non-coding RNAs (ncRNAs) are a class of RNA molecules that do not encode proteins. Studies show that ncRNAs are not only involved in cell proliferation, apoptosis, differentiation, metabolism and other physiological processes, but also involved in the pathogenesis of diseases. Cardiac remodeling is the main pathological basis of a variety of cardiovascular diseases. Many studies have shown that the occurrence and development of cardiac remodeling are closely related with the regulation of ncRNAs. Recent research of ncRNAs in heart disease has achieved rapid development. Thus, we summarize here the latest research progress and mainly the molecular mechanism of ncRNAs, including microRNAs (miRNAs), long non-coding RNAs (lncRNAs) and circular RNAs (circRNAs), in cardiac remodeling, aiming to look for new targets for heart disease treatment.

## 1. Introduction

Cardiovascular disease is the first leading cause of death which seriously threatens the health and quality of human life. Cardiac remodeling is the pathological process induced by the adaptive changes of cardiac insufficiency, and is closely related to the occurrence and development of many kinds of heart disease. Stress stimuli such as inflammation, pressure overload, oxidative injury and myocardial infarction (MI) can cause cardiac remodeling, the continued progress of which may ultimately develop to arrhythmia, heart failure, and even sudden death. Basically, the cardiac structure, function and phenotype have changed during cardiac remodeling, exhibiting as pathological cardiac hypertrophy with fetal gene re-expression, myocyte apoptosis, aging, necrosis, and extracellular matrix excessive fibrosis.

Cardiac remodeling involves complex molecular mechanisms. Thus, looking for key molecules that participate in the development process of cardiac remodeling is of great significance to elucidate the molecular mechanism, as well as to explore new ways of prevention and treatment of cardiovascular diseases. Non-coding RNAs (ncRNAs) are not only involved in cell proliferation, apoptosis, differentiation, metabolism and other important biological processes, but also closely related to the occurrence, development, treatment and diagnosis of diseases. In recent years, the research of ncRNAs in heart disease has been rapidly developed. More and more studies have revealed that ncRNAs play an important role in the development of cardiac remodeling. In this review, we focus on the latest research progress and mechanisms of ncRNAs, especially microRNAs (miRNAs), long non-coding RNAs (lncRNAs) and circular RNAs (circRNAs) in cardiac remodeling, aiming to find new therapeutic targets for heart disease treatment.

## 2. The Classification and Function of ncRNAs

ncRNAs are a class of RNA molecules that do not encode proteins and function directly at the RNA level. Based on the function, length and structure dissimilarity, ncRNAs can be divided into transfer RNAs (tRNAs), ribosomal RNAs (rRNAs), small nuclear RNAs (snRNAs), small nucleolar RNAs (snoRNAs), guide RNAs (gRNAs), miRNAs, piwi-interacting RNAs (piRNAs), small interfering RNAs (siRNAs), lncRNAs, and circRNAs [1]. Table 1 shows the length range and main functions of each class of ncRNAs. At present, studies are mainly focused on the role of miRNAs, lncRNAs, piRNAs and circRNAs in the normal growth and development of body, physiological functions and pathological processes.

**Figure 1:**
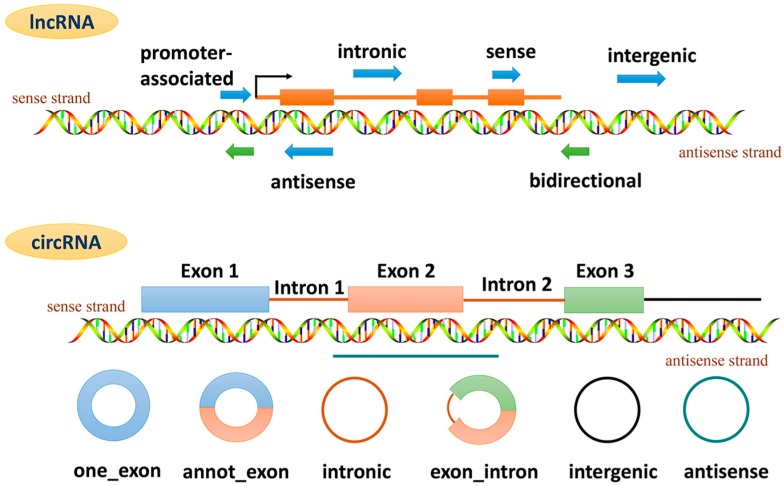
Long non-coding RNA (lncRNA) and circular RNA (circRNA) classification based on genomic location and context.

miRNAs are a class of endogenous single-strand ncRNA molecules with a length of 19–23 nucleotides, whose sequences are highly conserved among different species. Combined with the specific sites of the target mRNAs through base complementary, miRNAs can promote the degradation of mRNAs or inhibit the translation of mRNAs in the post transcriptional level, so as to exert its negative regulation on gene expression. Typically, an miRNA can regulate the expression of multiple genes, but a certain gene also can be precisely regulated by a plurality of miRNAs. lncRNAs, a class of RNA molecules more than 200 nucleotides long, generally do not encode proteins. According to their positions in the genome, lncRNAs can be classified into sense, antisense, intronic, intergenic, bidirectional and promoter-associated (Figure 1). lncRNAs have an mRNA-like structure, some with poly (A) tail, and show dynamic expression pattern and alternative splicing during differentiation. When compared with the coding gene, lncRNAs share less sequence conservation and show lower expression level [2]. lncRNAs can regulate gene expression in epigenetic, transcriptional and post-transcriptional levels, as well as directly modulate protein activity [3,4,5,6]. circRNAs are a class of RNA molecules derived from exon reverse splicing or intron lariat. Depending on their genomic structures, circRNAs can be divided into one_exon, annot_exon, intronic, exon_intron, intergenic and antisense (Figure 1). The expression of circRNAs is relatively stable because of their closed ring structure, which prevents them from being affected by RNA exonuclease, and is tissue- and developmental stage-specific [7]. circRNAs can play their regulatory function by competing endogenous RNA (ceRNA) mechanism, that is, acting as miRNA sponge in the cell [8]. In addition, circRNAs can regulate the linear splicing competition of pre-mRNAs and the transcription of parental genes [9,10]. piRNAs are small RNA molecules with a length of about 24–30 nucleotides. The investigation on piRNA is still at a preliminary stage, and current studies have found that piRNA binds to PIWI family proteins and forms a complex called piRNA-induced silencing complex (piRISC) which subsequently regulates the gene silencing pathway in germ cells [11].

## 3. miRNAs and Cardiac Remodeling

In recent years, miRNAs have been the hotspot of life science research and the most widely studied ncRNAs. At present, more than 3000 miRNAs have been identified in human genome [12], which are involved in regulating the expression of more than 50% functional genes. miRNAs play an important role in maintaining cell homeostasis. Dysregulation of miRNAs can lead to a large number of diseases [13], including a variety of pathological processes of heart disease [14,15].

Thus far, many miRNA molecules involved in the regulation of cardiac hypertrophy and fibrosis have been identified. Olson’s group firstly find that the cardiac specific miR-208 encoded by the intron of *α-MHC* (*myosin heavy chain 6*, *myh6*) gene is abnormally expressed in pressure-induced cardiac hypertrophy and fibrosis, with the transformation of *MHC* gene from the adult type (*α-MHC*, *myh6*) to the fetal type (*β-MHC*, *myh7*) [16]. Knockout of miR-208 can inhibit cardiac hypertrophy induced by coarctation of the aorta. On the other hand, the first identified negative regulator of cardiac hypertrophy is miR-133 [17]. Overexpression of miR-133 can inhibit the hypertrophic response induced by pressure overload, and this anti-hypertrophic effect is achieved by inhibiting the expression of its target genes *RhoA* (Ras homolog gene family, member A), *Cdc42* (cell division cycle 42) and *Nelf-A*/*WHSC2* (Negative elongation factor complex member A/ Wolf-Hirschhorn Syndrome Candidate 2). miR-29 is one of the best studied anti myocardial fibrosis factors. Van Rooij et al. [18] have found that the level of miR-29 was significantly reduced under stress, making its targets such as collagen, elastin, fibrin and other extracellular matrix protein synthesis increased, thereby promoting the process of myocardial fibrosis. Over-expression of miR-29 can inhibit the synthesis of collagen and resist myocardial fibrosis. Clearly, the molecular mechanism of miRNA regulation is solely the inhibition of target mRNA expression, but the downstream targets are various and related to multiple signaling pathways in all aspects of life activities. Table 2 summaries the roles of recent identified miRNAs and their target genes in several aspects of pathological cardiac remodeling, such as hypertrophy, fibrosis and apoptosis. Moreover, the interaction between miRNAs and other ncRNAs makes it more interesting and worthy of research, which will be discussed in the following parts.

## 4. lncRNAs and Cardiac Remodeling

**Figure 2:**
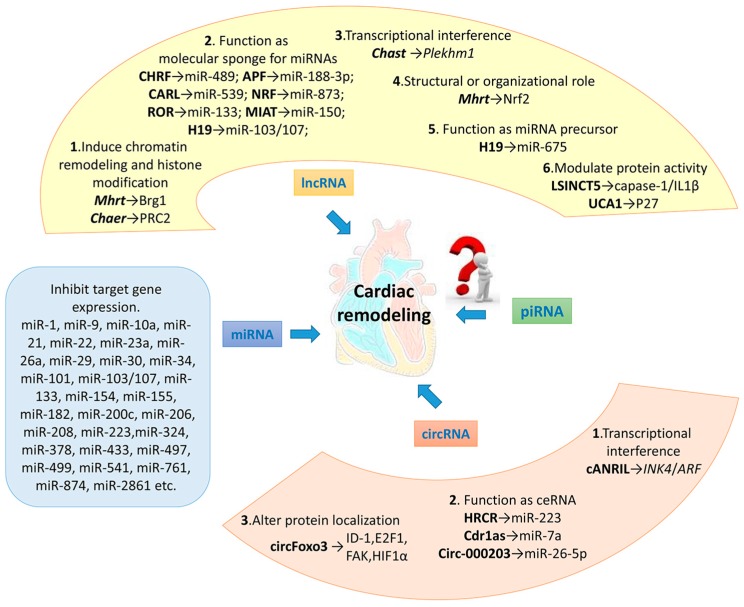
Summary of the ncRNAs and their molecular mechanisms in cardiac remodeling.

It is reported that only about 1.5% of the human genome can encode proteins, while the vast majority of the genome is in a state of non-transcribed or transcribes to ncRNAs [66]. As the next-generation high-throughput sequencing technology develops, a growing number of lncRNAs are identified. Thus far, a plurality of lncRNAs has been demonstrated to be involved in the regulation of cardiac remodeling via several different mechanisms (Figure 2). This makes lncRNAs function as potential therapeutic targets for the treatment of cardiac hypertrophy, heart failure and other cardiac disorders.

### 4.1. lncRNA and Epigenetic Regulation

#### 4.1.1. lncRNA and Chromatin Remodeling

In transverse aortic constriction (TAC)-induced pressure overload mice model, Han et al. [67] have indicated that cardiac-specific lncRNA *Mhrt*, which is the antisense transcript of myosin heavy chain 7 (*Myh7*), can exert its cardioprotective function through lncRNA-chromatin interaction mechanism. Pathological stress activates the chromatin remodeling factor Brg1 to form a complex composed of Brg1-HDAC-Parp, which directly binds to the promoter region of *Mhrt* and inhibits the transcription and expression of *Mhrt*. The helicase domain of Brg1 is capable of binding with both lncRNA *Mhrt* and chromatinized DNA targets, but not naked DNA. *Mhrt* inhibits the binding of Brg1 to its genomic DNA targets by competitively binding with helicase to prevent the occurrence of chromatin remodeling. *Mhrt* and Brg1 form a complete feedback circuit that plays a crucial role in the protection of the heart. Restored expression of *Mhrt* under pathological conditions can protect the heart from excessive hypertrophy and heart failure [67].

#### 4.1.2. lncRNA and Histone Methylation

In a recent study, a cardiac-enriched lncRNA named *Chaer* (cardiac-hypertrophy-associated epigenetic regulator) has been demonstrated to be both necessary and sufficient for the development of cardiac hypertrophy [68]. It was identified from the dysregulated lncRNAs in heart failure mice induced by pressure overload. The transcript of *Chaer* was found to be predominantly located in the nucleus and highly conserved among mice, rats and humans. In *Chaer*-KO mice, hypertrophy of the heart was significantly attenuated after TAC operation. Moreover, the pathological fibrosis was weakened and the heart function was protected. Mechanistically, a 66-mer motif in *Chaer* can directly interact with the enhancer of zeste homolog 2 (EZH2) subunit of polycomb repressor complex 2 (PRC2), and interfere with PRC2 targeting to genomic loci, thereby inhibiting histone H3 lysine 27 methylation in the promoter regions of cardiac hypertrophy-associated genes. The interaction between *Chaer* and PRC2 is transiently induced after hormonal or stress stimulation, and is a prerequisite for epigenetic reprogramming and induction of cardiac hypertrophy-associated genes. It is essential that the inhibition of *Chaer* expression in heart prior to, but not after, the onset of stress overload can significantly reduce cardiac hypertrophy and heart dysfunction. In addition, the level of the human transcript *CHAER* was significantly increased in dilated cardiomyopathy compared with that in normal hearts. In human induced pluripotent stem cell-derived cardiomyocytes (hiPSC-CMs), *CHAER* specially interacts with EZH2 and has rapamycin sensitivity. Overexpression of *CHAER* exhibits a similar hypertrophy phenotype with mouse homolog, indicating a cross-species regulatory function of *Chaer*.

### 4.2. lncRNA as Molecular Sponge for miRNA

The molecular mechanism of lncRNAs as ceRNAs in regulating cardiac remodeling has been extensively studied. In Ang II-induced hypertensive mice, cardiac hypertrophy related lncRNA named CHRF can inhibit the expression and activity of miR-489 as an endogenous molecular sponge, and subsequently up-regulate the level of myeloid differentiation factor88 (MyD88), which is a direct target of miR-489, to promote cardiac hypertrophy [36]. In cardiomyocytes treated with phenylephrine (PE), the expression level of lncRNA-ROR (or lincRNA-ST8SIA3) is significantly increased, knock-down of which reduces the hypertrophic response [69]. It is revealed that lncRNA-ROR functions as miR-133 sponge, and overexpression of miR-133 can reverse the pro-hypertrophic effect of lncRNA-ROR. In addition, lncRNA myocardial infarction associated transcript (MIAT) has been demonstrated to be a pro-hypertrophic regulator in cardiac hypertrophy induced by Ang II via acting as a molecular sponge for miR-150 [70]. 

Cardiomyocytes autophagy plays an important role in maintaining homeostasis, myocardial cell size, as well as cardiac structure and function. Wang et al. [54] have identified a lncRNA that can regulate autophagy in cardiac myocytes and named it autophagy promoting factor (APF). The results show that miR-188-3p can suppress autophagy-induced cardiomyocyte death or MI by targeting autophagy-related protein 7 (ATG7). APF acts as miR-188-3p sponge to regulate the level of ATG7 and thus plays a role in regulating autophagy and MI [54].

Myocardial cell death is the cytological basis of many cardiovascular diseases, inhibition of which can improve cardiac function. lncRNA plays an important role in ischemia-reperfusion (I/R) injury induced cardiac remodeling by participating in the regulation of myocardial apoptosis. A cardiac apoptosis-related lncRNA called CARL is able to upregulate the level of prohibitin 2 (PHB2) by competitively binding with PHB2 upstream negative regulator miR-539, thereby inhibiting cardiomyocyte apoptosis and MI injury-induced cardiac remodeling [62].

In the past, cell necrosis was thought to be a passive form of cell death. However, recent studies have demonstrated that certain types of necrosis are regulated by signaling pathways such as receptor-interacting serine/threonine-protein kinase (RIPK) 1 and 3. In the process of MI and hydrogen peroxide (H_2_O_2_)-induced myocardial necrosis, the levels of miR-103/107 are upregulated, which subsequently suppress the expression of Fas-Associated protein with Death Domain (FADD). FADD can interact with receptor-interacting protein 1 (RIP1) to inhibit the formation of RIP1/RIP3 complex, thereby promoting the development of myocardial necrosis. lncRNA H19 serving as the miR-103/107 sponge can reduce the endogenous miR-103/107 levels, inhibit the cell necrosis and MI size induced by ischemic injury, as well as the myocardial fibrosis and cardiac remodeling, thus improving cardiac function [53]. Additionally, a lncRNA named necrosis-related factor (NRF) has been revealed to participate in the regulation of cardiomyocytes programmed necrosis by directly targeting miR-873 and consequently suppressing the expression of RIPK1/RIPK3, which are miR-873 downstream targets [71].

### 4.3. lncRNA Regulates the Expression of Adjacent Protein Coding Gene

Viereck et al. [72] have identified a cardiac-specific, lncRNA molecule capable of promoting cardiac hypertrophy from differential lncRNA expression profiles in sham and TAC-operated mouse hearts, and named it *Chast* (cardiac hypertrophy-associated Transcript). Mechanistically, the hypertrophy promoting factor NFAT can bind to *Chast* promoter and activate the transcription of *Chast*. *Chast* plays an important role in regulating the level of its adjacent gene *Plekhm1*, which is an autophagy regulatory factor, thus blocking the autophagy of cardiomyocytes and promoting cardiac hypertrophy. Although lncRNAs are less conserved and have tissue-specificity compared to miRNAs, the researchers have observed high expression levels of the *Chast* human homolog *CHAST* in hypertrophic heart tissue of patients with aortic stenosis, suggesting that *CHAST* can be used as a drug target for the treatment of cardiac hypertrophy. It has also been demonstrated that silencing of *Chast* with antisense oligonucleotide inhibitors can alleviate cardiac hypertrophy induced by coarctation of the aorta and improve cardiac function.

### 4.4. lncRNA and Protein Complexes Bind to the Gene Promoter Region, Regulating Gene Expression

Doxorubicin (Dox) is one of the most effective broad-spectrum anticancer drugs. However, it can induce cardiotoxicity that promotes cardiomyocytes apoptosis and cardiomyopathy. As described above, lncRNA *Mhrt* is a heart protective regulator against pathological cardiac remodeling [67]. Recently, researchers have reported that obestatin can attenuate Dox-induced cardiac dysfunction via upregulating *Mhrt* expression [73]. Mechanistically, *Mhrt* can positively regulate the expression of Nrf2 (Nuclear factor erythroid 2-related factor 2), a signaling pathway which is involved in preventing cardiac remodeling and heart failure [73,74]. Further, they prove that *Mhrt* prompts combination of H3 histone and *Nrf2* promoter, thereby enhancing the transcription of *Nrf2* and increasing Nrf2 protein level.

### 4.5. lncRNA as miRNA Precursor

As one of the earliest discovered imprinted gene, lncRNA H19 has been shown to play an important role in mammalian embryogenesis and tumorigenesis. The first exon of H19 is able to encode miR-675. A novel study reveals that H19 functions as a negative regulator in pathological cardiac hypertrophy mediated by miR-675 [75]. Overexpression of H19 can reduce the increase of cell size and hypertrophy-relate gene levels induced by PE, whereas knock-down of H19 promotes cardiomyocyte hypertrophy. Moreover, miR-675 overexpression or knock-down reveals its inhibitive effects on myocardial hypertrophy. Then rescue and mutation experiments were performed to demonstrate that miR-675 can mediate the function of H19 to inhibit cardiomyocyte hypertrophy. Finally, Ca^2+^/calmodulin-dependent protein kinase II δ-isoform (CaMKIIδ) has been identified to be a direct target of H19/miR-675 axis, which is involved in the regulation of cardiac hypertrophy. These findings reveal a new pathway: H19 exerts its cardiomyocyte hypertrophy negative regulatory role as miR-675 precursor, the mature form of which directly inhibits the expression of CaMKIIδ.

### 4.6. lncRNA Modulates Protein Activity

A recently identified lncRNA, Urothelial carcinoma-associated 1 (UCA1), is reported to be an anti-apoptosis regulator, the levels of which are markedly decreased in cardiac I/R injury rat models and cardiomyocytes treated with H_2_O_2_ [76]. Knock-down of UCA1 reduces the viability of cardiomycytes and promotes cell apoptosis. Mechanistically, UCA1 can directly suppress p27 at the protein level, while overexpression of p27 activates the caspase-3 apoptotic pathway. Taken together, the precise molecular mechanism is that downregulation of UCA1 in primary cardiomyocytes exhibits pro-apoptotic function partially through stimulating p27 protein expression, revealing the important role of UCA1 in I/R injury induced cardiac remodeling. Similarly, lncRNA LSINCT5 is involved in the regulation of myocardial apoptosis induced by B-type-natriuretic peptide (BNP) via activating the capase-1/interleukin (IL)-1β signaling pathway [77].

### 4.7. Others

lncRNA BC088254 was identified from differentially expressed lncRNA microarray data in cardiac hypertrophic rats and was upregulated after TAC. Bioinformatics and coexpression network analyses suggest that lncRNA BC088254 has a relationship with phb2 and can weakly affect the expression of phb2, but the result still needs to be verified [78]. Two lncRNAs named MI-associated transcript 1 (MIRT1) and 2 (MIRT2) are revealed to be significantly increased in MI mice and relevant to multiple genes known to be involved in cardiac remodeling, such as *Icam1*, *Tgfb1*, etc. [79]. Furthermore, through gain and loss of function investigations, cardiac fibroblast-enriched lncRNAs n379599, n379519, n384648, n380433 and n410105 are indicated to participate in the TGF-β signaling pathway to regulate the fibrosis related genes expression in ischemic cardiomyopathy [80]. At the late stage of diabetes, hearts undergo hypertrophy and other remodeling events, which lead to heart dysfunction and failure eventually. Zhang et al. [81] report that downregulation of lncRNA metastasis-associated lung adenocarcinoma transcript 1 (MALAT1) can inhibit the apoptosis of cardiomyocytes and improve heart function in diabetic rats. On the contrary, another team proves that MALAT1 is not necessary during pressure overload-induced cardiac remodeling and failure in mice [82]. These findings suggest the specific functions of lncRNAs and the importance of validating the proposed lncRNAs in relevant disease models.

## 5. circRNAs and Cardiac Remodeling

As early as the 1990s, scientists had discovered the existence of circRNAs [83]. However, circRNAs were once considered to be produced by RNA splicing errors or splicing process by-products due to technique and approach limitations at that time. In 2013, it was revealed that circRNAs function as molecular sponge for miRNAs [8], making circRNAs become a new hotspot in the field of ncRNAs after miRNAs and lncRNAs.

Due to the advanced sequencing technology and data analysis methods, a growing number of circRNAs have been uncovered from human and murine hearts [84,85,86]. The first circRNA profiling study has identified 575 circRNA species from adult murine hearts and showed that several circRNAs are relevant for the cardiovascular disease pathogenesis [85]. The comparative analyses of the circRNA expression from rats (neonatal and adult), mice (sham or after TAC) and humans (failing, non-failing) have detected more than 9000 candidate circRNAs from each species [86]. Recently, a detailed annotation and analysis of genome-wide circRNA expression uncovered 15,318 cardiac circRNAs in the human heart [84]. All of these provide a good basis for studying the role of circRNAs in heart disease.

### 5.1. circRNA Regulates the Adjacent Gene Expression

Although the functional research on circRNA is still in its infancy, emerging evidence suggests that circRNAs possess important biological functions and have a close relationship with cardiovascular diseases. Genome wide association studies (GWAS) have shown that the single nucleotide polymorphisms (SNPs) in 9p21.3, the neighboring chromosome sequence of *INK4*/*ARF* gene, are associated with susceptibility to atherosclerotic vascular disease (ASVD). CircRNA cANRIL is the antisense transcript of *INK4*/*ARF* gene. It can regulate the expression of *INK4*/*ARF* and increase the risk of ASVD [87].

### 5.2. circRNA Functions as ceRNA

One important functionary mechanism of circRNAs is as molecular sponges for miRNAs. Wang et al. have identified a heart-related circRNA, HRCR, which functions as molecular sponge of miR-223 and protects the heart from pathological hypertrophy [33]. This is a new signal pathway, which is composed of HRCR/miR-223/ARC, participating in the regulation of cardiac hypertrophy and heart failure. Results show that miR-223 transgenic mice can spontaneously undergo cardiac hypertrophy and heart failure, while miR-223 knockout mice can resist the pathological cardiac hypertrophy induced by isoprenaline (Iso). miR-223 can suppress the expression of the anti-apoptotic protein ARC through binding to its gene 3′ untranslated region. In general, HRCR can adsorb the endogenous miR-223, then inhibit its function and up-regulate the expression of its downstream target ARC, thus playing a role in inhibiting pathological cardiac hypertrophy [33]. In a similar study, circRNA Cdr1as, which is the antisense of cerebellar degeneration-related protein 1 transcript, functions as miR-7a sponge and is proved to be able to promote apoptosis and MI development in heart [88]. Overexpression of miR-7a can protect cardiomyocytes from hypoxia-induced apoptosis by directly inhibiting the apoptotic process related target genes PARP and SP1. Both in vitro and in vivo experiments illustrate that overexpression of Cdr1as aggravates MI injuries by sponging miR-7a and thus upregulating the levels of PARP and SP1, while miR-7a co-overexpression significantly attenuates the changes Cdr1as-induced [88]. Recently, circRNA_000203 has been identified from cardiac fibroblasts to have the pro-fibrosis effect as miR-26-5p sponge, thus blocking the interactions between miR-26-5p and its target fibrosis-associated genes Col1a2 and CTGF [89]. These proved pathways consisting of HRCR/miR-223/ARC, Cdr1as/miR-7a/ SP1 and circRNA_000203/miR-26-5p/ Col1a2, CTGF in cardiac myocytes and fibroblasts provide new therapeutic targets for the treatment of cardiac remodeling related diseases.

### 5.3. circRNA Binds to Specific Proteins, Altering Their Cellular Localization

Yang et al. [90] have reported that circRNA circ-Foxo3, encoded by the forkhead box transcription factor 3 (Foxo3) and mainly localized in the cytoplasm, is able to interact with and suppress the aging-associated proteins, resulting in increased severity of cardiomyopathy. Circ-Foxo3 is. In the model of cardiomyopathy induced by Dox, overexpressed circ-Foxo3 can interact with the anti-senescent protein ID-1, transcription factor E2F1 and the anti-stress proteins FAK, HIF1α, which are in turn restricted to be translocated to the nucleus to exert their functions, leading to cardiac senescence and apoptosis; whereas silencing endogenous circ-Foxo3 inhibits senescence and relieves cardiomyopathy [90]. Later, they demonstrate that the oral *Ganoderma* spore oil possesses cardiovascular protective effect through regulating circ-Foxo3 expression [91]. Evidence reveals that *Ganoderma* treatment can suppress the expression level of circ-Foxo3, reduce left ventricular hypertrophy and improve cardiac function generated by TAC. Despite all of this, the regulatory mechanism of circRNAs and their role in cardiac remodeling remain to be fully discovered.

## 6. Conclusions and Prospective

Evidence for miRNAs as important disease regulating factors has been well developed. It has given great expectation to using miRNAs as therapeutic drug targets. Thus far, clinical trials have been carried out on several miRNA candidates to treat tumor disease. For instance, inhibitor of miR-122 (Miravirsen) for the treatment of hepatitis C has entered the second phase of clinical trial, and mimics of miR-34a (MRX34) are being tested in a phase I clinical trial in patients with hepatocellular carcinoma or hepatic metastases [92,93]. More and more miRNAs are demonstrated to regulate cardiac remodeling, but there is still no reports regarding miRNAs as drug targets used in the treatment of cardiovascular diseases. Thus, it is undoubtedly very promising for the application of miRNAs in cardiovascular disease diagnosis, treatment and prognosis. Nevertheless, in-depth understanding of the mechanism of miRNAs in the development of cardiovascular diseases will still be the focus of future research. Recently, Wang et al. [94] have revealed that the excessive reactive oxygen species (ROS) in cardiac myocytes can modified miR-184 to oxidized miR-184, which promotes apoptosis by suppressing the expression of its non-native targets Bcl-xL and Bcl-w and increases the susceptibility of the heart to I/R injury. Indeed, this novel mechanism of ROS in regulating heart disease offers a new research angle for miRNAs.

As new regulatory molecules, the mechanisms of lncRNAs and circRNAs are more complex than that of miRNAs. For instance, they function as molecular scaffolds to regulate gene expression by binding with transcription factors or chromatin remodeling complex proteins, act as mRNA and miRNA endogenous sponges, participate in epigenetic regulation by directly binding to gene promoter region, or regulate mRNA stability of the neighboring genes and so on. Similarly, further in-depth research of lncRNAs and circRNAs in heart diseases, especially the mechanism investigation, is urgently requested. Remarkably, as an important class of small RNA molecules, the function of piRNA is gradually being recognized. Current studies have revealed that piRNA mainly plays a role in germ cells. However, few studies have reported on somatic cells, even the relationship between piRNA and heart disease, which is a new field well worth exploring.

## Figures and Tables

**Table 1:** The classification and function of non-coding RNAs.

ncRNAs	Length	Main Functions
tRNAs	74~95 nt	Transfer amino acids to ribosomes during protein synthesis
rRNAs	121~5000 nt	Ribosome components, directly involved in the synthesis of proteins in robosome
snRNAs	100~300 nt	Process mRNA precursor (splicing and maturation)
snoRNAs	100~300 nt	Guide chemical modifications of other RNAs, such as rRNAs, tRNAs and snRNAs
gRNAs	55~70 nt	Participate in RNA editing
miRNAs	19~23 nt	Negatively regulate gene expression by promote mRNA degradation or inhibit mRNA translation
piRNAs	24~30 nt	Play roles in gametogenesis, maintain transposon silencing, translation suppression, epigenetic regulation
siRNAs	21~25 nt	Silence complementary target mRNA
lncRNAs	>200 nt	Regulate gene expression in epigenetic, transcriptional, post-transcriptional levels, miRNA sponge
circRNAs	Circular	As competing endogenous RNA or miRNA sponge; regulate alternative splicing and parental gene expression

**Table 2:** Effects of miRNAs in pathological cardiac remodeling.

miRNAs	Effects	Target Genes/ Signaling Pathway	Pathological Conditions	Reference
**Hypertrophy**				
let-7a	Anti-	*calmodulin*	Ang II	[19]
miR-1	Anti-	*HADC4*	Thyroid hormone	[20]
miR-9	Anti-	*myocardin*	Iso and Aldo	[21]
miR-10a	Anti-	*Tbx5*	Ang II, TAC	[22]
miR-22	Anti-	*PURB*, *Sirt1*, *Hdac4*	Pressure overload	[23,24]
miR-23a	Pro-	*MuRF1*	Iso and Aldo	[25]
miR-26a	Anti-	*GATA4*	Ang II, TAC	[26]
miR-34	Pro-	*VEGF*, *vinculin*, *POFUT1*, *Notch1*, *SEMA4B*	TAC	[27]
miR-101	Anti-	*Rab1a*	Ang II, TAC	[28]
miR-155	Pro-	*Jarid2*	TAC, activated calcineurin Tg	[29]
miR-182	Pro-	*Bcat2*, *Foxo3*, *Adcy6/*Akt	PIGF	[30]
miR-206	Pro-	*Foxp1*	YAP, pressure overload	[31]
miR-212/132	Pro-	*Foxo3*	Pressure overload	[32]
miR-223	Pro-	*ARC*	Iso, TAC	[33]
miR-365	Pro-	*Skp2/* mTOR	Ang II	[34]
miR-378	Anti-	*MAPK1*, *IGFR1*, *GRB2*, *KSR1/*MAPK	TAC	[35]
miR-489	Anti-	*MyD88*	Ang II, TAC	[36]
miR-497	Anti-	*Sirt4*	Ang II, TAC	[37]
miR-541	Anti-	-	Ang II	[38]
**Fibrosis**				
miR-1	Anti-	*Fibullin-2/*MAPK	Pressure overload	[39]
miR-21	Pro-	*Spry1/*ERK-MAP kinase *PTEN/*SMAD7	Pressure overload, Ang II	[40,41]
miR-22	Anti-	*TGFβRI*	Ang II	[42]
miR-30	Anti-	*CTGF*	Renin-2 Tg	[43]
miR-34a	Pro-	*Smad4*	MI	[44]
miR-101	Anti-	*c-Fos/*TGF-β1	Ang II, CAL	[45]
miR-154	Pro-	*Atg7*	TAC	[46]
miR-200c	Pro-	*DUSP-1/*MAPK	High glucose	[47]
miR-433	Pro-	*AZIN1*, *JNK1/*TGFβ/Smad3	MI	[48]
**Apoptosis/Necrosis**				
miR-30	Anti-	*p53*, *Cyclophilin D*	H_2_O_2_, I/R	[49,50]
miR-34a	Pro-	*PPP1R10*, *ALDH2*	MI	[51,52]
miR-103/107	Pro-	*FADD*	I/R, H_2_O_2_	[53]
miR-188-3p	Anti-	*Atg7*	I/R, A/R	[54]
miR-324-5p	Anti-	*Mtfr1*	I/R, A/R	[55]
miR-361	Pro-	*PHB1*	M/I, H_2_O_2_	[56]
miR-378	Anti-	*Caspase-3*	CAL/Hypoxia	[57]
miR-421	Pro-	*Pink1*	I/R, H_2_O_2_	[58]
miR-484	Anti-	*Fission 1*	I/R, Anoxia, Dox	[59]
miR-499	Anti-	*CnAα*, *CnAβ*	I/R, Anoxia	[60]
miR-532-3p	Pro-	*ARC*	Dox	[61]
miR-539	Pro-	*PHB2*	I/R, Anoxia	[62]
miR-761	Anti-	*MFF*	I/R, H_2_O_2_	[63]
miR-874	Pro-	*Caspase-8*	I/R, H_2_O_2_	[64]
miR-2861	Pro-	*ANT1*	I/R, H_2_O_2_	[65]

## Abbreviations

Adcy6	Adenylate cyclase 6
ALDH2	Aldehyde dehydrogenase 2
Aldo	Aldosterone
AMPK	AMP-activated protein kinase
Ang II	Angiotensin II
ANT1	Adenine nucleotide translocase 1
APF	Autophagy promoting factor
A/R	Anoxia/reoxygenation
ASVD	Atherosclerotic vascular disease
Bcat2	Branched chain amino acid transaminase 2
BNP	B-type-natriuretic peptide
CAL	Coronary artery ligation
calcineurin-NFAT	Calcineurin-nuclear factor of activated T cells
CaMKIIδ	Ca^2+^/calmodulin-dependent protein kinase II δ-isoform
CARL	Cardiac apoptosis related lncRNA
Cdc42	Cell division cycle 42
Cdr1as	Antisense of cerebellar degeneration-related protein 1
ceRNA	Competing endogenous RNA
*Chaer*	Cardiac-hypertrophy-associated epigenetic regulator
*Chast*	Cardiac hypertrophy-associated Transcript
CHRF	Cardiac hypertrophy related lncRNA
circRNAs	Circular RNAs
CnA	Calcineurin catalytic subunit
CTGF	Connective tissue growth factor
Dox	Doxorubicin
EZH2	Zeste homolog 2
FADD	Fas-Associated protein with Death Domain
Foxo3	Forkhead box transcription factor 3
Foxp1	Forkhead box protein P1
GATA4	GATA Binding Protein 4
GRB2	Growth factor receptor-bound protein 2
GWAS	Genome wide association studies
HADC4	Histone Deacetylase 4
hiPSC-CMs	Human induced pluripotent stem cell–derived cardiomyocytes
H_2_O_2_	Hydrogen peroxide
HRCR	Heart-related circRNA
IGFR-1	Insulin-like growth factor receptor 1
IL	Interleukin
I/R	Ischemia-reperfusion
Iso	Isoprenaline
JAK-STAT	Janus Kinase- Signal transducers and activators of transcription
Jarid2	Jumonji, AT rich interactive domain 2
KSR1	Kinase suppressor of ras 1
lncRNAs	Long non-coding RNAs
MALAT1	Metastasis-associated lung adenocarcinoma transcript 1
MAPKs	Mitogen-activated protein kinases
MFF	Mitochondrial fission factor
MI	Myocardial infarction
MIAT	Myocardial infarction associated transcript
miRNAs	MicroRNAs
MuRF1	Muscle specific ring finger protein 1
MyD88	Myeloid differentiation factor88
*Myh7*	Myosin heavy chain 7
ncRNAs	Non-coding RNAs
Nelf-A	Negative elongation factor complex member A
NF-κB	Nuclear factor-κB
NRF	Necrosis-related factor
PE	Phenylephrine
PHB2	Prohibitin 2
PI3K/Akt	Phosphatidylinositol 3-kinase/Akt
PIGF	Placental growth factor
Pink1	PTEN Induced Putative Kinase 1
piRISC	piRNA-induced silencing complex
piRNAs	Piwi-interacting RNAs
PKC	Protein kinase C
POFUT1	Protein *O*-fucosyltranferase 1
PPP1R10	Protein Phosphatase 1 Regulatory Subunit 10
PRC2	Polycomb repressor complex 2
PTEN	Phosphatase And Tensin Homolog
PURB	Purine-rich element binding protein B
Rab1a	Ras-related protein Rab-1A
RhoA	Ras homolog gene family, member A
RIPK	Receptor-interacting serine/threonine-protein kinase
ROS	Reactive oxygen species
rRNAs	Ribosomal RNAs
SEMA4B	Semaphorin 4B
siRNAs	Small interfering RNAs
Sirt4	Sirtuin 4
Skp2	S-Phase Kinase-Associated Protein 2
SMAD7	SMAD Family Member 7
snoRNAs	Small nucleolar RNA
SNPs	Single nucleotide polymorphisms
snRNAs	Small nuclear RNAs
Spry1	Sprouty homolog 1
TAC	Transverse aortic constriction
Tbx 5	T-box 5
Tg	Transgenic
tRNAs	Transfer RNAs
TNF-α	Tumor necrosis factor-α
UCA1	Urothelial carcinoma-associated 1
VEGF	Vascular endothelial growth factors
WHSC2	Wolf-Hirschhorn Syndrome Candidate 2
YAP	Yes-associated protein
